# Nanoherbicides for Efficient, Safe, and Sustainable Weed Management: A Review

**DOI:** 10.3390/nano15171304

**Published:** 2025-08-24

**Authors:** Fangyuan Chen, Pengkun Niu, Fei Gao, Zhanghua Zeng, Haixin Cui, Bo Cui

**Affiliations:** Institute of Environment and Sustainable Development in Agriculture, Chinese Academy of Agricultural Sciences, Beijing 100081, China; 82101235292@caas.cn (F.C.); 821012430095@caas.cn (P.N.); zengzhanghua@caas.cn (Z.Z.); cuihaixin@caas.cn (H.C.)

**Keywords:** nanoherbicide, carrier encapsulation, efficiency, safety, weed management

## Abstract

Weeds are a significant factor affecting crop yield and quality. Herbicides have made crucial contributions to ensuring stable and high grain production, but the low effective utilization rate and short duration of traditional formulations have led to excessive application and a range of ecological and environmental issues. Nanoherbicides, particularly carrier-coated systems, can simultaneously leverage the small size, large specific surface area, and high permeability of nanoparticles, as well as the multifunctionality of carriers, to synergistically enhance the efficacy and safety of the formulations. This provides a scientific and promising strategy for overcoming the functional deficiencies of traditional formulations. Nevertheless, there are currently relatively few articles that systematically review the research progress and performance advantages of nanoherbicides. This review provides a concise overview of the preparation methods and structural characteristics of nanoherbicides. It primarily highlights the classification of carrier-coated nanoherbicides, along with representative studies and their distinctive properties across various categories. Based on this foundation, the performance advantages of nanoherbicides are systematically summarized. Finally, the major challenges and future prospects in this research field are proposed. This review offers valuable insights and methodological guidance for the design and rational application of efficient, environmentally friendly nanoherbicides.

## 1. Introduction

With the global population projected to reach 9.7 billion by 2050, it is imperative that crop yields increase by over 70% to satisfy the anticipated demand, thus posing a substantial challenge to the food supply [1]. Meanwhile, climate change-induced disasters, such as droughts, floods, and extreme heat, have further exacerbated the issue of food security [2]. As a critical component of the farmland ecosystem, weeds not only influence crop growth but also serve as a primary cause of reduced agricultural yields. Global crop yield losses attributed to weeds reach up to 31.5%, resulting in annual economic damages estimated at approximately USD 32 billion [3]. As agrochemicals designed to eliminate or suppress weed growth, herbicides have consistently dominated the largest share of the global pesticide market. In 2023, global pesticide consumption was ranked as follows: herbicides > fungicides > insecticides, with respective usage amounts of 1732.3 metric tons, 816.38 metric tons, and 757.54 metric tons [4]. However, the effective utilization rate of traditional pesticide formulations remains suboptimal, with over 70% of active ingredients being lost to the environment through pathways such as drift, volatilization, and leaching [5]. When the development of novel herbicide compounds lags, the prolonged and excessive application of herbicides to maintain control efficacy not only disrupts biodiversity and soil ecosystems, but also intensifies weed resistance [6], and even induces various human diseases through food chain contamination [7]. Therefore, developing high-efficiency and environmentally friendly formulations has become an urgent need to enhance the effectiveness and functionality of herbicides.

In recent years, the emergence of nanotechnology has introduced new opportunities for sustainable agricultural practices, offering significant potential to facilitate the transformation from traditional farming methods to precision and smart agriculture [8]. The interdisciplinary convergence of nanomaterials and nanotechnologies with pesticide formulation science has spurred the robust advancement of nanopesticides. In 2019, nanopesticides were recognized as the top emerging technology in the field of chemistry by the International Union of Pure and Applied Chemistry. The term “nanopesticides” encompasses pesticide formulations that either intentionally incorporate entities within the nanometer scale (typically less than 500 nm, as regulated by the European Union and the US Environmental Protection Agency), or is purported to exhibit novel properties attributable to their small size [9,10]. A comprehensive statistical analysis reveals that when compared with non-nanoscale analogs, nanopesticides exhibit a 31.5% enhancement in overall efficacy against target organisms, including an 18.9% increased efficacy in field trials. Additionally, the premature loss of active ingredients prior to reaching target organisms is diminished by 41.4%, paired with a 22.1% lower leaching potential in soil. Particularly, the toxicity of nanopesticides to non-target organisms is reduced by 43.1% [10]. Although the advantages of nanopesticides have been extensively demonstrated, current research and development efforts are predominantly focused on insecticides and fungicides [11,12], with relatively few reports on nanoherbicides. On the one hand, since certain typical herbicides, such as paraquat, glufosinate ammonium, and glyphosate are water-soluble and exist in a molecular state in water, they do not require nano-processing from the perspective of dispersion. On the other hand, due to differences among insecticides, fungicides, and herbicides in terms of target organisms, application methods, and pricing, greater attention has been directed toward nanoinsecticides and nanofungicides.

The construction of nanoherbicides should not only emphasize efficacy but also take into account their distinctive properties, including high application dosage, risks to non-target organisms, and environmental impacts, with a particular focus on the safety assessment. For example, although paraquat exhibits low cost and broad-spectrum herbicidal activity, its high toxicity to humans with a lethal dose of 35 mg/kg for adults and established association with an increased risk of Parkinson’s disease have resulted in its prohibition in numerous countries [13]. Furthermore, herbicides such as 2,4-dichlorophenoxyacetic acid (2,4-D), glyphosate, atrazine, sulfentrazone, and metribuzin have also been identified as water pollutants [14].

Nanoherbicides are gaining increasing attention due to their high utilization efficiency and minimal residual characteristics. Nevertheless, reviews that systematically summarize their research advancements and functional attributes are still limited. This review outlines the preparation methodologies of nanoherbicides and categorizes them based on carrier types. It comprehensively analyzes the distinctive properties of various nanoherbicide formulations, summarizes their demonstrated advantages in agricultural applications, and further proposes forward-looking perspectives on future development. This review provides inspiration and guidance for the design and rational application of efficient and green nanoherbicides.

## 2. Preparation Methods of Nanoherbicides

The preparation methodologies for nanoherbicides are analogous to those developed for nanoinsecticides and nanofungicides, primarily categorized into two distinct approaches: direct nanoparticulation and nanoencapsulation technology. Nanoparticulation refers to the process of directly converting pesticides into nanoparticles, which can be accomplished through either top-down or bottom-up approaches [15]. The top-down method involves the gradual fragmentation of bulk materials into nanoparticles through mechanical or physical processes such as wet milling, high-pressure homogenization, melt emulsification, and ultrasonic dispersion. The bottom-up strategy synthesizes nanoparticles by assembling molecules including microprecipitation, solvent evaporation, interfacial polymerization, etc. [16,17]. Cheng et al. fabricated a quinclorac nanosuspension with a particle size of 200 nm via wet milling technology, exhibiting superior herbicidal efficacy against barnyard grass at merely 50% of the dosage required for commercial wettable powder [18]. Kumar et al. employed ultrasonic emulsification to prepare a nanoemulsion by encapsulating atrazine with clove oil, achieving an encapsulation efficiency of up to 95% and a 1.5-fold enhancement in herbicidal activity compared to wettable powder [19].

**Figure 1:**
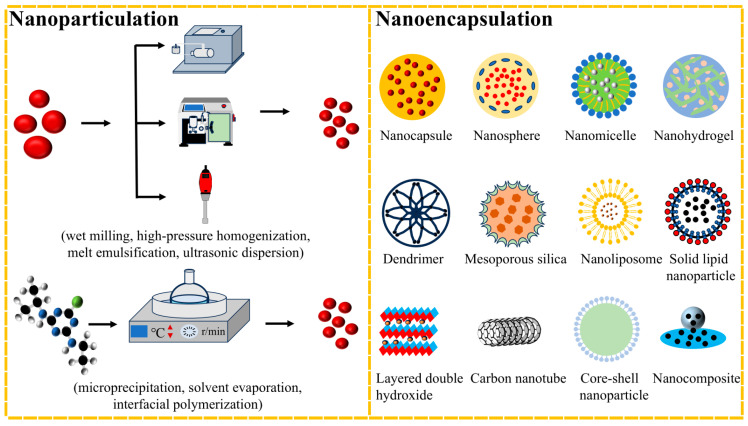
Preparation strategies of nanoherbicides.

Nanoencapsulation technology involves encapsulating active ingredients within carrier materials to enhance pesticide stability, reduce loss, and enable stimuli-responsive release through structural regulation and functional modification of carriers. This strategy is capable of significantly enhancing pesticide utilization rate while prolonging duration. The methods for loading active ingredients into carriers include adsorption, coupling, coating, and inlaying. Figure 1 illustrates the different preparation methods of nanoherbicides. In comparison to non-carrier-coated nanoherbicides, carrier-based nanoherbicide systems have received greater attention and research focus. This is primarily because carrier encapsulation can significantly enhance pesticide stability and enable controlled release. Additionally, the modifiability of carrier materials offers multi-dimensional regulatory flexibility in system design, thereby facilitating better adaptation to complex field application requirements. The size, morphology, crystal structure, and chemical composition of nanomaterials all significantly influence the physicochemical properties of pesticide delivery systems [20]. Therefore, the selection of carrier materials should not only consider their pesticide-loading capacity, but also balance various factors, including biodegradability, environmental safety, processability, and chemical compatibility with active ingredients [21]. This review categorizes carrier-coated nanoherbicides based on their carrier types and provides a comprehensive explanation of their characteristics. Major carrier-coated nanoherbicides and their properties are presented in Table 1.

## 3. Types and Characteristics of Nanoherbicides

### 3.1. Polymer-Based Nanoherbicides

**Figure 2:**
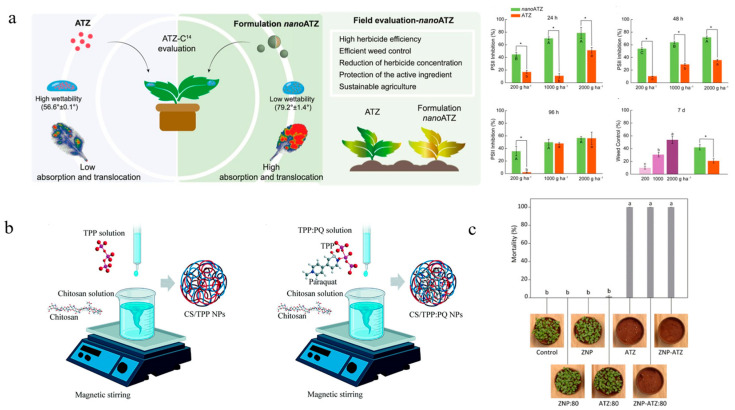
Polymer-based nanoherbicides: (**a**) Performance and field control efficacy of atrazine@PCL nanocapsules [23]. Copyright, 2021 Elsevier; (**b**) Preparation of paraquat@chitosan/TPP nanoparticles [91]. Copyright, 2021 Royal Society of Chemistry; (**c**) Atrazine@zein herbicidal activity of nanocapsules [49]. Copyright, 2023 Royal Society of Chemistry.

Polymeric materials can be categorized into natural, synthetic, and semi-synthetic polymers. Predominant polymer carriers utilized in nanoherbicide systems comprise PCL, Polyethylene glycol, TPP, chitosan, zein, alginate, lignin, cellulose, and polyethylenimine. PCL has excellent biocompatibility [82], and the formed nanoparticles exhibit remarkable colloidal stability and high encapsulation capacity [83,84]. Diyanat et al. developed Pretilachlor-loaded PCL nanocapsules with an encapsulation efficiency as high as 99.5%. The nanocapsules enhanced the herbicidal activity against barnyard grass, while reducing their toxicity to non-target cells [37]. Fraceto’s team synthesized a series of PCL-based nanoherbicides and carried out a comprehensive investigation into the fabrication protocols, herbicidal efficacy, residue effects, interaction mechanisms with target organisms, and impacts on non-target species. The atrazine@PCL nanocapsules exhibited excellent stability and reduced soil mobility and genotoxicity. They demonstrated 2 to 10 times higher herbicidal efficacy against target plants (*Brassica juncea*, *Amaranthus viridis*, and *Bidens pilosa*) compared to traditional formulations, while showing no significant physiological effects on soybean (Figure 2a) [23]. Investigations into the interaction mechanisms with target organisms revealed that atrazine@PCL initially adhered to the leaf surface of *Brassica juncea*, followed by translocation through the vascular tissues into cells, where it degraded chloroplasts to exert herbicidal activity [26]. In addition, neither pre-emergence nor post-emergence treatment with atrazine@PCL induced adverse effects on the growth of maize [85]. Metribuzin@PCL showed reduced retention and mobility in soil compared to commercial formulations, while enhancing delivery efficiency to target organisms [29,30]. Diyanat et al. prepared metribuzin@PCL nanocapsules, which not only enhanced the herbicidal activity against *Portulaca oleracea* but also reduced its soil mobility and environmental negative impacts [28]. Kannamreddy et al. encapsulated sulfentrazone using polyethylene glycol as a carrier, improving its herbicidal activity while minimizing adverse effect on soil and groundwater [34]. Mahmoudian et al. encapsulated haloxyfop-R-methyl within poly (methyl methacrylate) using emulsion polymerization technique, extending its release duration to six days [35]. Tang et al. constructed a 168 nm-sized 2,4-D/branched poly (ethylene imine) self-assembly through noncovalent molecular recognition. The surfactant-free self-assembled nanoparticles with improved physicochemical properties including strong positive charges, reduced volatilization rate, low surface tension, and decreased leaching potential exhibited control efficacy comparable to that of the 2,4-D sodium salt form containing tween 80 [51].

A major drawback of synthetic polymers lies in their slow degradation rate, whereas natural polymers are increasingly favored in pharmaceutical and pesticide fields owing to their eco-friendliness, biocompatibility, and availability [86,87]. Chitosan, a natural deacetylated compound, is the second-most-abundant biopolymer after cellulose [88] and exhibits potential resistance against a broad spectrum of bacterial, fungal, and viral pathogens [89,90]. Khan et al. developed nanoherbicides using chitosan as the carrier for either the sole encapsulation of mesosulfuron methyl or the co-loading of mesosulfuron methyl, florasulam, and 2-methyl-4-chlorophenoxyacetic acid, aiming to enhance herbicidal activity [40]. Chitosan-composite nanoparticles are formed by combining chitosan with other compounds, achieving efficient delivery through performance complementarity and optimization. Ghaderpoori et al. fabricated a paraquat-loaded nanohydrogel using chitosan, xanthan, and TPP, which significantly enhanced the adhesion and efficacy of the herbicide [41]. Sodium alginate is a natural polysaccharide with excellent stability, solubility, viscosity, and safety required for excipients in pharmaceutical formulations. Babaei et al. constructed the chloridazon-loaded alginate/chitosan nanocapsules via ionic gelation method, which extended the release duration of chloridazon and reduced the pesticide dosage [42]. Pontes et al. developed the chitosan/TPP composite nanoparticles loaded with paraquat. It was found that the amount of lipid peroxidation, photooxidizable P700 reaction center content, and NADPH/NADP^+^ ratio levels were significantly decreased in spinach leaf tissue exposed to the nanoherbicides compared to those with the non-encapsulated herbicide (Figure 2b) [91]. Maruyama et al. fabricated two types of nanoparticles by employing chitosan/alginate (377 nm) and chitosan/TPP (478 nm) as carriers for the co-encapsulation of imazapic and imazapyr, showing minimal effects on soil microbial communities and genotoxicity compared to traditional formulations [44]. Grillo et al. encapsulated paraquat through the cross-linking of chitosan with TPP, thereby reducing its soil adsorption and genotoxicity in both Chinese hamster ovary cells and onion cells [92,93]. Rashidipour et al. delivered paraquat using a composite carrier system comprising pectin, chitosan, and TPP, enhancing its herbicidal efficacy against *Brassica juncea* while effectively reducing pesticide residues in soil and toxicity to mammalian lung cells [45]. Owing to the functional groups and inherent positive charges within its molecular structure, chitosan also functions as an ideal coating material for core–shell systems. Grillo et al. coated atrazine@PCL nanocapsules with chitosan and demonstrated that the shell thickness influenced both the particle size and the release profile of atrazine [38]. Sousa et al. developed an atrazine@PCL@chitosan nanosystem with an encapsulation efficiency exceeding 90%, demonstrating that the nanoherbicide increased PSII activity inhibition by 96% compared to uncoated atrazine [39]. Artusio et al. prepared sodium alginate hydrogels encapsulating hydrophilic dicamba via the inverse miniemulsion template method, which prolonged the release period of dicamba [47].

Zein is a group of alcohol-soluble proteins extracted from corn gluten meal and classified as Generally Recognized as Safe by the US Food and Drug Administration. Bragança et al. prepared zein-based atrazine-loaded nanocapsules via the antisolvent precipitation method, achieving an encapsulation efficiency exceeding 90%. These nanocapsules enhanced herbicidal activity against *Brassica juncea* and reduced the soil mobility of atrazine (Figure 2c) [49]. Heydari et al. constructed a tribenuron-methyl@zein nanosystem with an encapsulation efficiency of 81%, which prolonged the herbicide release time and enabled a 50% reduction in dosage under the same control efficacy [50]. Munhoz-Garcia et al. constructed three nanoherbicides loaded with glyphosate using chitosan/TPP, zein/poloxamer, and zein/lignin as carriers, respectively. Among these, the glyphosate@zein/poloxamer system exhibited the highest stability, enhanced herbicidal activity against *Amaranthus hybridus*, with no toxic effects on Roundup Ready [46].

Despite their notable advantages in biosafety, natural polymers still exhibit certain limitations, including immunogenic reactions, uncontrollable degradation rates, and inadequate mechanical strength, which may result in premature release and degradation of pesticides in actual application [94]. In contrast, synthetic polymers offer superior stability and structural controllability, making them preferred carriers for long-term release applications and harsh environmental conditions. However, their potential toxicity and high production costs remain critical considerations. Although polymers exhibit excellent biocompatibility and do not directly harm crops or the environment, some polymeric materials lack sufficient properties to carry corresponding active pharmaceutical molecules. This limitation can only be addressed by developing and conjugating them with appropriate trigger groups (e.g., photodegradable, enzyme-degradable, pH-controlled release groups, etc.) [95]. Therefore, based on the environmental requirements, cost constraints, and performance demands of the target application scenario, the development of composite carriers through the integration of diverse polymer properties presents a promising future direction for herbicide delivery.

### 3.2. Clay-Based Nanoherbicides

Clay, a natural silicate mineral, serves as an excellent matrix in pesticide formulations due to its low cost and easy availability. Clay nanocomposites, defined as composites formed by exfoliating or embedding nanoparticles into clay matrices, are typically synthesized via solution-blending, in situ intercalative polymerization, and melt blending techniques [96]. These nanocomposites can effectively enhance the solubility and dispersion of poorly water-soluble pesticides. By encapsulating active ingredients within the matrix, they also extend the shelf life and release duration of active ingredients. LDH, halloysite, montmorillonite, and attapulgite are typical clay-based carriers.

**Figure 3:**
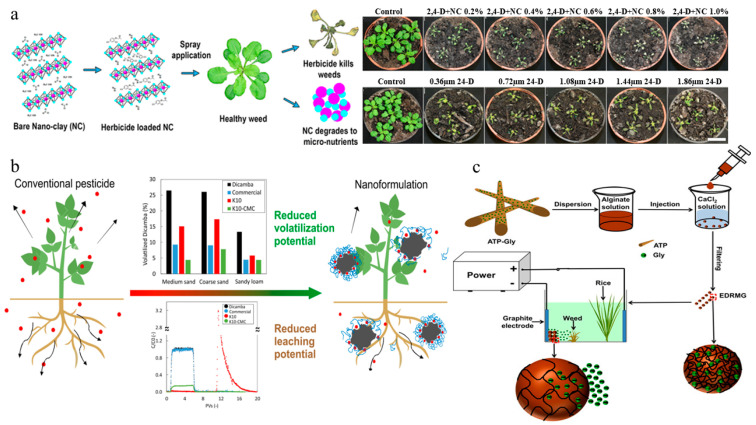
Clay-based nanoherbicides: (**a**) 2,4-D-loaded MgAl-LDH nanoclay system and its herbicidal activity against *Arabidopsis thaliana* [52]. Copyright, 2019 American Chemical Society; (**b**) Volatilization and leaching performances of dicamba loaded in K10 montmorillonite [55]. Copyright, 2022 Elsevier; (**c**) Electrically driven release of glyphosate in gel-based nanocomposites [57]. Copyright, 2020 American Chemical Society.

LDH is a type of anionic clay composed of nanoscale octahedral sheets stacked via co-precipitation of constituent elements [97]. Nadiminti et al. synthesized a 2,4-D-loaded MgAl-LDH nanoclay system, improving herbicidal activity against *Arabidopsis thaliana* compared to traditional formulations (Figure 3a) [52]. Ghazali et al. fabricated a nanohybrid herbicide by inserting 2,4,5-trichlorophenoxybutyrate and 2-methyl-4-chlorophenoxy acetate as dual guests into ZnAl-LDH through an ion-exchange process. The two active ingredients exhibited differential release rates and their release time was extended by approximately 4000 min [53]. Khatem et al. constructed imazamox@LDH (anionic clay) and imazamox@Cloisite10A (cationic clay) systems, which exhibited comparable characteristics in terms of release profiles, herbicidal activity, and environmental compatibility [54].

Halloysite clay is a naturally occurring aluminosilicate. Zhong et al. encapsulated atrazine within the lumen of halloysite nanotubes and subsequently incorporated them into a polyvinyl alcohol/starch composite material, successfully achieving the controlled release of atrazine. The cumulative release amount over 96 **h** reached only 61% [56]. Montmorillonite is a kind of cheap and abundant natural layered-silicate clay, exhibiting strong adsorption properties due to its large specific surface area and high charge capacity [98]. Granetto et al. adsorbed dicamba onto K10 montmorillonite and then coated it with carboxymethyl cellulose, yielding a system with excellent controlled-release properties and stability, while reducing volatilization and leaching of the herbicide (Figure 3b) [55]. Zha et al. constructed a pH-responsive delivery system by encapsulating glyphosate- and dopamine-modified attapulgite within sodium alginate hydrogel, which mitigated the degradation, adsorption, and leaching of the active ingredient in soil [58]. Zhang et al. produced a nanohydrogel system composed of attapulgite, glyphosate, and calcium alginate for electrical-driven release and migration, demonstrating favorable biocompatibility with fish and mice. (Figure 3c) [57].

The adsorption properties of clay enable them to not only serve as delivery carriers for herbicides but also remove pesticide residues in water. For example, modified montmorillonite nanoclay can effectively remove 2,4-D from water [99]. Furthermore, Jia et al. incorporated glufosinate-loaded halloysite into poly (butylene adipate-*co*-terephthalate)/poly (lactic acid) mulch films. This integration not only maintained the excellent mechanical properties of the mulch films but also imparted controlled-release herbicidal functionality [100]. This investigation expands the application scenarios of nanoherbicides.

Nanoclay materials exhibit remarkable versatility in various scientific and industrial fields due to their excellent adsorption capacity, structural reinforcement properties, and multifunctional potential. The cytotoxicity of nanoclay materials varies depending on the type of clay, concentration, and experimental conditions [101]. Although studies have shown that LDH has low toxicity, its extensive properties in promoting plant growth and soil improvement have attracted more attention from researchers than other metal nanoparticles [102].

### 3.3. Silica-Based Nanoherbicides

**Figure 4:**
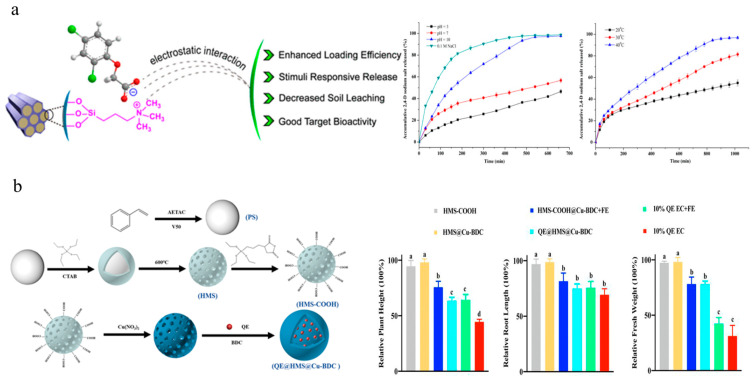
Silica-based nanoherbicides: (**a**) Preparation and controlled release of 2,4-D sodium salt@MSN-trimethylammonium nanoparticles [67]. Copyright, 2018 American Chemical Society; (**b**) Preparation and safety experiments of quizalofop-p-ethyl@HMS@Cu-BDC and rice safety [69]. Copyright, 2023 John Wiley & Sons Ltd.

Silica is the second-most-abundant element in the earth’s crust and is considered a beneficial nutrient due to its promoting role in plant growth [103]. MSN have an ordered pore structure, adjustable pore size, high specific surface area, excellent mechanical properties and thermal stability, as well as high biocompatibility [104,105]. Cao et al. utilized functionalized MSN as carriers to independently develop nanoherbicides for encapsulating 2,4-D sodium salt and diquat, which improved the pesticide-loading capacity and herbicidal activity (Figure 4a) [67,68]. HMS possess large internal cavities, three-dimensionally ordered pore arrangements, and tunable surface functional groups [106]. Deng et al. deposited ultra-thin films of copper-benzene-1,4-dicarboxylic acid metal–organic frameworks onto the surface of carboxylated HMS as gatekeepers for quizalofop-*p*-ethyl. This system demonstrated highly efficient efficacy on both sensitive and resistant barnyard grasses, with superior absorption, transportation, and ACCase activity inhibition performance compared to emulsifiable concentrate (Figure 4b) [69]. Ji et al. adopted a pesticide–fertilizer combination strategy to achieve controlled and targeted delivery of agrochemicals. This system consisted of three components: (1) HMS for encapsulating 2,4-D and 1-tetradecanol; (2) polydopamine coating to provide a photothermal effect; (3) a zeolitic imidazolate framework to provide micronutrient Zn^2+^ and encapsulate dinotefuran. This system achieved synergistic effects of weeding and insecticide and nutrient supply [73]. Wang et al. functionalized silica microspheres with cinnamidite and then encapsulated them with γ-cyclodextrin to form pendimethalin-loaded bilayer microspheres. This approach effectively prolonged the release time of pesticides and reduced genotoxicity [71]. Although silica-based nanomaterials are beneficial for improving pesticide utilization efficiency, target-specific absorption, and partially exerting plant nutrient functions, their bioaccumulation effects and ecological risks still require further verification. Additionally, the large-scale, low cost, and environmentally friendly production of high-purity silica-based nanomaterials remains a challenge.

### 3.4. MOFs-Based Nanoherbicides

**Figure 5:**
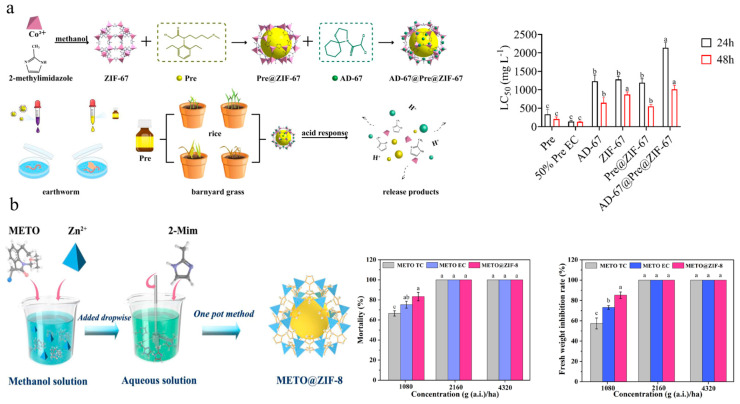
MOFs-based nanoherbicides: (**a**) Preparation of AD-67@Pretilachlor@ZIF-67 and safety of earthworms [77]. Copyright, 2023 Royal Society of Chemistry; (**b**) Metolachlor@ZIF-8 preparation and herbicidal activity [74]. Copyright, 2022 John Wiley & Sons Ltd.

MOFs are crystalline porous materials constructed with metal-ion clusters as central nodes, coordinated with one or more organic ligands through coordination bonds [107,108]. Currently, the applications of MOF in agriculture mainly involve wastewater treatment [109,110,111], sensors [112], and pesticide delivery [113,114]. The structures of MOFs are diverse and highly tunable and they have unique advantages including high surface area, high-temperature stability, accelerated adsorption/desorption kinetics, and biocompatibility. Mejías et al. achieved one-step encapsulation of ortho-disulfides in functionalized zinc MOF, resulting in over 80% inhibition of root growth against *Lolium perenne*, *Echinochloa crus-galli*, and *Amaranthus viridis* [72]. Guo et al. utilized ZIF-67 to load Pretilachlor and subsequently adsorbed the safener 4-(dichloroacetyl)-1-oxa-4-azospiro [4,5] decane (AD-67) onto the MOF surface, thus constructing an AD-67@Pretilachlor@ZIF-67 controlled-release system. Compared with traditional formulations, AD-67@Pretilachlor@ZIF-67 was more prone to adhering to plant surface, thereby enhancing herbicidal activity (Figure 5a) [77]. Ren et al. constructed a metolachlor@ZIF-8 controlled-release system that did not affect maize plant growth and significantly reduced the risk of metolachlor-induced phytotoxicity (Figure 5b) [74]. Compared with other porous materials, MOFs exhibit greater flexibility and diversity in both composition and structure [115]. Sierra-Serrano et al. developed a Cu^2+^-based MOF system loaded with glufosinate ammonium, which demonstrated excellent antibacterial (against *Staphylococcus aureus* and *Escherichia coli*) and herbicidal activities, as well as biocompatibility [75]. Lee et al. constructed an atrazine@MOF-5/polyvinyl alcohol/starch delivery system which achieved controlled release, reduced the risks and runoff and leaching of herbicide, and provided a warming and moisture-retaining effect for plants [76]. MOFs, serving as adsorbents for removing herbicides from water, are also a significant focus of research in agricultural applications. The maximum adsorption capacity of HUiO-66s for glyphosate was as high as 400 mg/g [116]. MIL-101-NH_2_ exhibited an adsorption capacity of 348.5 mg/g for 2,4-D under the condition of pH = 7 [117]. However, the practical application of MOFs in agriculture still faces several challenges, such as high production costs, stability, scalability, and compatibility under different environmental conditions [118]. Meanwhile, heavy metals in MOF structures, like chromium and nickel, may accumulate in the environment, posing potential risks. COF, which are also porous crystalline materials, are distinct from MOFs in that their structural backbones consist exclusively of organic building blocks without metal nodes [119,120]. Deng et al. constructed a cyhalofop-butyl-loaded COF system, which effectively delivered cyhalofop-butyl into the leaves and stems of weeds, thereby enhancing the herbicidal activity against *Echinochloa crus-galli* and *Leptochloa chinensis* [81].

### 3.5. Carbon-Based Nanoherbicides

**Figure 6:**
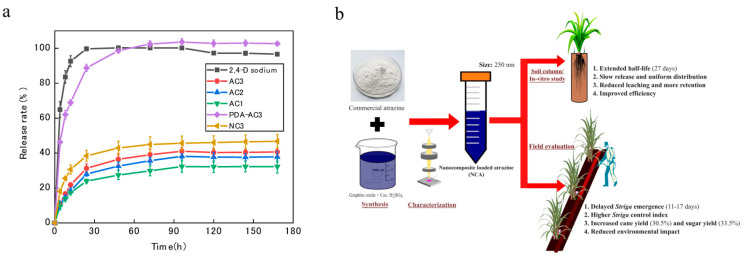
Carbon-based nanoherbicides: (**a**) Release profiles of 2,4-D sodium from different activated carbon-based delivery systems [79]. Copyright, 2019 Multidisciplinary Digital Publishing Institute; (**b**) Synthesis, characterization, and evaluation of nanocomposite atrazine for *Striga* control [78]. Copyright, 2024 Springer Nature.

Biochar has become an ideal choice for agrochemicals delivery due to its low cost, eco-friendliness, stability, and modifiable properties [121,122]. Yang et al. utilized activated carbon as carriers to construct five types of 2,4-D sodium nanoherbicides, all of which demonstrated favorable adsorption and sustained-release properties (Figure 6a) [79]. Iyarin et al. synthesized a graphene oxide-based nanoherbicide loaded with atrazine, which prolonged the release of atrazine and improved its herbicidal activity and biocompatibility (Figure 6b) [78]. Tang et al. co-assembled amphiphilic-cationic carbon dots (CPC-CDs) with acifluorfen through noncovalent interactions to obtain the stable fluorescent nanoparticles (ACI@CPC-CDs). Under low light intensity, CPC-CDs can be applied as the internal light source to promote the formation of more singlet oxygen to damage the leaf cell membrane, consequently improving the herbicidal activity of acifluorfen [80].

## 4. Stimuli-Responsive Nanoherbicides

**Figure 7:**
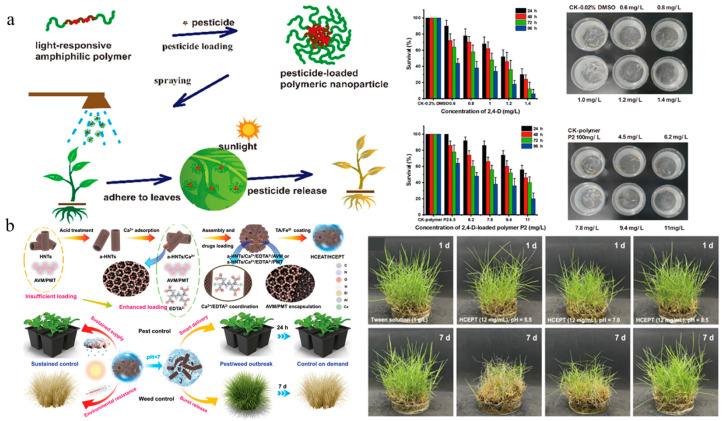
Stimuli-responsive nanoherbicides: (**a**) The release mechanism of 2,4-D nanoparticles and their biosafety on zebrafish [127]. Copyright, 2022 American Chemical Society; (**b**) Preparation and herbicidal effect of pH-responsive nanoherbicide system co-loading avermectin and prometrinet [66]. Copyright, 2024 John Wiley & Sons Ltd.

The normal growth of crops requires suitable environmental conditions including temperature, light, soil pH, and moisture. However, although these growth factors contribute to crop development, they can also create favorable conditions for weed proliferation and pathogen reproduction, thereby imposing dual constraints on crop yield [123]. Stimuli-responsive delivery systems can release pesticides in response to environmental stimuli (light, temperature, pH, enzyme, redox potential, and ion concentration). These controlled-release systems can not only improve the pesticide utilization rate and reduce waste and environmental pollution, but also enable precise delivery of active ingredients, which are conductive to reducing impact on non-target organisms [124,125]. Given the increasing demand for improving the efficacy and safety of herbicides, stimuli-responsive nanoherbicides have progressively emerged as a cutting-edge and focal point in this field. The representative stimuli-responsive nanoherbicide systems are listed in Table 2. Chen et al. developed a light-responsive controlled-release system for glyphosate delivery by using photoisomerization of azobenzene and increased adhesion to weed leaves [126]. Shan et al. synthesized a biodegradable and photoresponsive amphiphilic polymer for the encapsulation of 2,4-D. The 2,4-D-loaded polymer nanoparticles showed excellent herbicidal activity and reduced toxicity to non-target organisms (Figure 7a) [127]. A temperature-responsive controlled-release herbicide with a core–shell structure was developed by Chi et al. using a nanocomposite consisting of attapulgite, NH_4_HCO_3_, amino silica oil, polyvinyl alcohol, and glyphosate [128]. Xiao et al. designed a nano-cocrystal material composed of clopyralid and phenazine, which intelligently modulated herbicide release according to multiple environmental factors, including temperature, pH, and soil inorganic salts. Importantly, it improved the foliar wettability and adhesion and increased the inhibition rate against Medicago sativa and Oxalis corniculata by about 27% with lower genotoxicity [129]. Teng et al. employed halloysite clay nanotubes to self-assemble into microspheres for the encapsulation of abamectin and prometryn and subsequently coated their surfaces with tannic acid and iron. This system not only demonstrated pH-responsive controlled-release properties, superior UV resistance, and enhanced leaf adhesion, but also significantly reduced the leaching effect (Figure 7b) [66]. Dong et al. encapsulated paraquat in a porous carbon–chitosan composite system to obtain a dual-responsive nanoherbicide, which accelerated pesticide release under acidic pH and high-temperature conditions, ensuring herbicidal efficacy while reducing cytotoxicity [130]. Liang et al. prepared a urease-responsive system by crosslinking isocyanate-functionalized silica with polyethyleneimine, which enhanced the thermal stability and photostability of pendimethalin. The pesticide release rate was positively correlated with temperature, and the rates under weakly acidic and weakly alkaline conditions were higher than that under neutral conditions, exhibiting longer duration and higher herbicidal activity [70]. Although stimuli-responsive nanoherbicides have demonstrated satisfactory controlled-release characteristics under laboratory conditions, in complex and variable farmland environments, precise design and optimization of response mechanisms and functional structures to ensure stable operation remain a significant challenge. In addition, the preparation of such herbicide-loading systems often encounters issues such as complex procedure, high cost, and unstable product reproducibility, which hinder their industrialization process.

## 5. Advantages of Nanoherbicides

**Figure 8:**
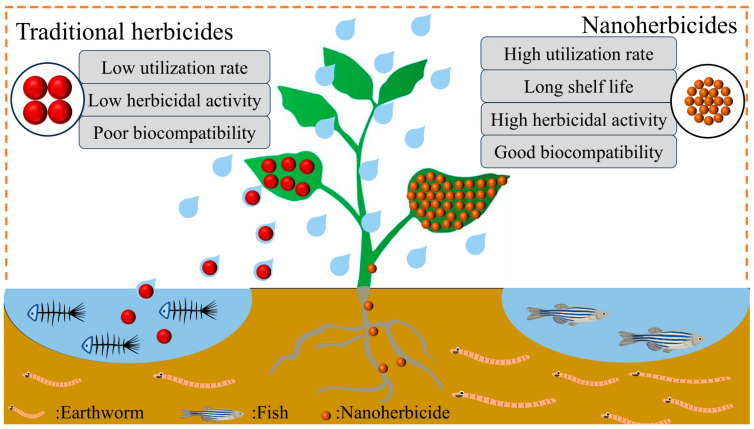
Performance differences between traditional herbicides and nanoherbicides.

Based on the literature reports, the advantages of nanoherbicides can be summarized as follows (Figure 8): (1) nano-sizing improves the solubility and dispersibility of poorly soluble herbicides in water; (2) the nanoencapsulation effect of carriers enhances the stability of active ingredients, extends the duration, reduces the dosage and frequency of herbicides, and decreases toxicity to non-target organisms and environmental pollution; (3) precise regulation of the composition, structure, and function of nanocarriers can effectively improve the leaf wetting, adsorption, and retention properties of pesticide droplets, impart environmental responsiveness to the nano-delivery system, and thereby enhancing the targeting, utilization rate, and bioactivity of herbicides.

## 6. Risks and Challenges of Nanoherbicides

### 6.1. Risks to the Ecological Environment

Although nanotechnology holds broad application prospects in agriculture, the current safety risk assessment of agricultural nanoparticles is severely insufficient, especially regarding their behavior, toxicity, and ecological interactions in agricultural environments, which remain unclear [136]. As a crucial component of nanoherbicides, nanocarriers’ ecotoxicological effects and bioaccumulation behavior are core elements in evaluating their environmental safety. In ecosystems, plants are producers and a key factor as the primary trophic level in the food chain. The accumulation of nanoparticles usually reduces plant transpiration and photosynthetic rates, decreases seed germination, growth, and root elongation, inhibits root water conductivity, and affects plant growth, thereby altering plant physiological processes [137,138]. Additionally, nanoparticles can exhibit indirect toxicological effects by leaching from soil into surface water and groundwater. The interactions between nanocarriers and soil microbial cells may lead to specific changes in microbial cell physiology and gene expression, thereby reducing the diversity and abundance of specific microbial populations in the soil [139,140]. Researchers have found that the accumulation of nanoparticles in plants starts with root adsorption, followed by distribution in plant tissues through modification processes such as crystal phase dissolution, biotransformation, and bioaccumulation. Both plant roots and above-ground parts can act as “hosts” for nanoparticles, which are absorbed and accumulated by plant cells [141,142]. Huang et al. found that artificially synthesized plant root exudates can promote the dissolution of copper nanoparticles and increase the bioavailability of free Cu^2+^ [143]. The combined accumulation of nanocarriers with other pollutants (such as heavy metals and pesticides) may produce synergistic toxicity, exacerbating harm to ecosystems.

The particle size, morphology, surface charge, and unique physicochemical characteristics of nanoherbicides may have unexpected impacts on crops, agricultural products, and ecosystems, further posing potential risks to humans [144]. The fate of nanoparticles in soil is comprehensively influenced by soil type, the intrinsic properties of nanoparticles, and environmental conditions. Among these, heavy soils (such as those with high clay content, rich organic matter, and heavy texture) significantly regulate the fate of nanoparticles due to their unique physicochemical properties. The strong retention and transformation capabilities of heavy soils for nanoparticles make them a “buffer zone” for nanoparticle pollution, reducing the risk of migration to groundwater or over long distances. However, they may also lead to the long-term accumulation of nanoparticles in surface soils, increasing the risk of biological exposure. Nanoparticles can enter the soil through precipitation, deposition in the form of dust and aerosols, direct soil absorption of gaseous compounds, leaf abscission, or human activities [145]. Once released into the agricultural environment, nanoparticles immediately undergo numerous transformations, which promote their accumulation in the soil. Soil properties or components, such as pH, organic matter, and water content, can mediate the dissolution process of metal-based nanoparticles and serve as potential sources of free metal ions. Zhang et al. proposed combining existing nutrient cycling and crop productivity models with nanoinformatics methods to optimize targeting, absorption, transportation, nutrient capture, and long-term effects on soil microbial communities, aiming to design nanoscale agrochemicals with optimal safety and functionality [146]. In the future, it will be necessary to combine material engineering (such as designing low toxicity, easily degradable nanocarriers) and environmental monitoring technologies to establish a more comprehensive risk assessment system, ensuring the safe and controllable application of nanotechnology in agriculture.

### 6.2. Challenges of Commercial Application

Currently, the commercial production of nanoherbicides still faces multiple challenges. Firstly, the high production cost and complex manufacturing processes result in low profits and economic returns for nanoherbicides in agricultural applications, which is a crucial reason hindering their commercialization [147]. Secondly, most studies on the performance of nanoherbicides have been conducted under laboratory conditions; however, in complex field environments, their performance is difficult to maintain stability due to the influence of dynamic factors such as temperature, humidity, ultraviolet radiation, and crop surface properties [148]. Thirdly, there is a lack of unified definitions and regulatory frameworks for nanoherbicides across different countries, which easily leads to the emergence of “pseudo-nano” products, causing market chaos and being detrimental to the healthy development of the nanopesticide industry [149]. Fourthly, the mechanisms underlying the synergistic effect and emission reduction in nanoherbicides remain unclear, and there is a lack of scientific evaluation methods, making it difficult to accurately assess their potential risks to the environment and human health. This may, to a certain extent, restrict their widespread application in environment-sensitive agricultural areas [150]. These challenges highlight the urgent need to conduct more sufficient field validations, establish standardized regulatory systems, and carry out comprehensive safety assessments to ensure that nanoherbicides can become an effective component in the integrated weed management of agricultural production.

## 7. Conclusions and Outlooks

Given the prevalence of herbicide resistance and relatively slow pace of new herbicide compound development, enhancing the effective utilization and efficacy of existing herbicides has become an increasingly viable strategy, with greater emphasis placed on innovations in formulation processing technologies. The small size, large specific surface area, high permeability of nanoparticles, and the multifunctionality of carriers make nanoherbicides an innovative strategy to overcome the functional deficiencies of traditional formulations. Extensive research has confirmed that nanoherbicides can improve the dispersibility and stability of poorly soluble herbicides, prolong the duration, and enhance the effective utilization rate and efficacy of active ingredients, while simultaneously reducing toxicity to non-target organisms and environmental pollution. This offers a promising avenue for sustainable agricultural development. Nevertheless, nanoherbicides still face some challenges in terms of production and application. (1) Safety assessment. In-depth research is needed on the mechanisms of migration and accumulation of nanoherbicides in the soil–plant system, as well as their interactions with non-target organisms, in order to establish a scientific risk assessment framework and clarify the thresholds for their long-term impacts on the ecosystem. (2) Targeting precision. At present, the reported herbicide delivery systems have weak capabilities in recognizing subcellular structures or specific molecular targets. Integrating nanotechnology with gene editing technology to develop novel herbicides with high targeting and selectivity will serve as a pivotal strategy for enhancing weed control efficacy in the field and improving safety. (3) Optimization of environmental response mechanisms. Elucidating the release kinetics and action modes of nanoherbicides under varying environmental parameters is required. A delivery system that synergistically responds to multiple factors should be designed to enhance their applicability in complex farmland scenarios. (4) Interdisciplinary integration. By leveraging the interdisciplinary integration of materials science, plant physiology, and artificial intelligence to develop degradable nanocarriers and dynamic monitoring technologies, it is promising to achieve intelligent matching between herbicide release and the growth cycle of weeds, thus substantially improving pesticide utilization rate and reducing ineffective losses. The future development of nanoherbicides will advance in the direction of “precision, greenness, and intelligence”. This not only helps to improve the efficiency and sustainability of agricultural production but also provides strong support for ensuring food security.

## Figures and Tables

**Table 1:** Carrier-coated nanoherbicides and their characteristics.

Carrier Materials	Carrier Types	Material Preparation Method	Active Ingredient	Performance	References
Poly(ε-caprolactone) (PCL)	Polymer	Preformed polymer interfacial deposition method	Atrazine	High herbicidal activity and biosecurity	[22,23,24,25,26,27]
PCL	Polymer	Preformed polymer interfacial deposition method	Metribuzin	High herbicidal activity and low environmental risk	[28,29,30,31]
Poly(lactic-*co*-glycolic acid)	Polymer	Solvent evaporation method	Atrazine	Slow release	[32,33]
Polyethylene glycol	Polymer	Solvent evaporation method	Sulfentrazone	High herbicidal activity and good biocompatibility	[34]
Poly(methyl methacrylate)	Polymer	Emulsion Polymerization method	Haloxyfop-R-methyl	High biocompatibility	[35]
PCL and lignin	Polymer	Preformed polymer inter-facial deposition method	Metribuzin	High herbicidal activity and good ecological compatibility	[36]
PCL	Polymer	Preformed polymer inter-facial deposition method	Pretilachlor	High herbicidal activity and low cytotoxicity	[37]
PCL and chitosan	Polymer	Preformed polymer inter-facial deposition method	Atrazine	Adjustable size and dispersion	[38,39]
Chitosan	Polymer	Ion gel method	Mesosulfuron methyl and florasulam and 2-methyl-4-chlorophenoxyacetic acid	Low toxicity and high herbicidal activity	[40]
Chitosan	Polymer	Ion gel method	Paraquat	Improved adhesion and herbicidal activity	[41]
Paraquat	Polymer	Ion gel method	Chloridazon	Controlled release	[42]
Chloridazon	Polymer	Ion gel method	Paraquat	Reduced soil adsorption and high herbicidal activity	[43]
Paraquat	Polymer	Ion gel method	Imazapic and imazapyr	High herbicidal activity and low genotoxicity	[44]
Imazapic and imazapyr	Polymer	Ion gel method	Paraquat	High herbicidal activity and reduced adsorption	[45]
Paraquat	Polymer	Interfacial polymerization and ionic gel method	Glyphosate	High biocompatibility and low toxicity	[46]
Glyphosate	Polymer	Reverse microemulsion template method	Dicamba	Prolonged release	[47]
Dicamba	Polymer	Coating method	Chloridazon and etribuzin	Reduction in migration in soil	[48]
Chloridazon and etribuzin	Polymer	Anti-solvent precipitation method	Atrazine	High herbicidal activity	[49]
Atrazine	Polymer	Anti-solvent precipitation method	Tribenuron-methyl	High herbicidal activity	[50]
Tribenuron-methyl	Polymer	Self-assembly method	2,4-D	Reduction in soil leaching	[51]
2,4-D	Clay	Ion exchange method	2,4-D	High herbicidal activity	[52]
2,4-D	Clay	Ion exchange method	2,4,5-Trichlorophenoxybutyrate and 2-methyl-4-chlorophenoxy acetate	Controlled release	[53]
2,4,5-Trichlorophenoxybutyrate and 2-methyl-4-chlorophenoxy acetate	Clay	Direct synthesis and co-precipitation method	Imazamox	Reduction in soil leaching	[54]
Imazamox	Clay	Direct adsorption method	Dicamba	Improved stability	[55]
Dicamba	Clay	Casting method	Atrazine	Prolonged release and reduced soil leaching	[56]
Atrazine	Clay	Ion gel method	Glyphosate	Controlled release and good biocompatibility	[57,58]
Glyphosate	Clay	Starch modification method	Atrazine	Prolonged release	[59]
Atrazine	Clay	Starch modification method	Alachlor	Prolonged release	[60]
Alachlor	Clay	Starch gel method	Ametryn	Extend the shelf life	[61]
Ametryn	Clay	In situ method	Glyphosate	Prolonged release, high utilization, and erosion resistance	[62]
Glyphosate	Clay	Starch modification method	Isoproturon	Long shelf life and reduced soil leaching	[63]
Isoproturon	Clay	Modification method	Mesotrione	Controlled release	[64]
Mesotrione	Clay	Immersion method	Amitrole	Controlled release	[65]
Amitrole	Clay	Self-assembly method	Prometryn.	Controlled release and reduced soil leaching	[66]
Prometryn	Silica	Grafting method	2,4-D sodium salt	High herbicidal activity and safety	[67]
2,4-D sodium salt	Silica	Grafting method	Diquat	Controlled release and high herbicidal activity	[68]
Diquat	Silica	Hard template method	Quizalofop-p-ethyl	High herbicidal activity	[69]
Quizalofop-p-ethyl	Silica	Interfacial polymerization method	Pendimethalin	Stable, controlled release, high herbicidal activity, and low genotoxicity	[70]
Pendimethalin	Silica	Crosslinking method	Pendimethalin	Controlled release and low genotoxicity	[71]
Pendimethalin	Metal–organic framework (MOF)	In situ method	DiS–NH2 and DiS-O-acetyl	High herbicidal activity	[72]
DiS–NH2 and DiS-O-acetyl	MOF	Hard template method	2,4-D	High herbicidal activity	[73]
2,4-D	MOF	One-pot method	Metolachlor	Controlled release, high herbicidal activity, and safety	[74]
Metolachlor	MOF	Ion ligand method	Glufosinate ammonium	High herbicidal activity and low genotoxicity	[75]
Glufosinate ammonium	MOF	Electrostatic spraying method	Atrazine	Low risk and extended release time.	[76]
Atrazine	MOF	Self-assembly method	Pretilachlor	High adhesion and herbicidal activity	[77]
Pretilachlor	Carbon	Hummer’s method	Atrazine	High herbicidal activity and biocompatibility	[78]
Atrazine	Carbon	Modification method	2,4-D sodium salt	Long shelf life	[79]
2,4-D sodium salt	Carbon	Electrostatic adsorption method	Acifluorfen sodium	Reduction in soil leaching	[80]
Acifluorfen sodium	COF	Electrostatic adsorption method	Cyhalofop-butyl	High herbicidal activity	[81]

**Table 2:** Stimuli-responsive nanoherbicides.

Stimulation Conditions	Responsive Materials	Active Ingredient	Response Release Rate	References
Light	Perylene-3-ylmethanol	2,4-D	90% release rate achieved in 40 h	[131]
Light	Azobenzene	Glyphosate	Achieved 100% release rate in 2.5 h	[126]
Light	Cinnamamide	Pendimethalin	The release rate in 72 h exceeds 80%	[71]
Light	1,1,3,3-Tetramethylguanidine	2,4-D	The release rate after 10 min reached 97.3%	[127]
Light	*o*-Nitrobenzyl dithiol and diacrylates	2,4-D	7 min release rate reaches 95.4%	[132]
Light	Coumarin	2,4-D	The release rate reaches 90% in 500 min	[133]
Light	Azobenzene	Paraquat	Reach 75% release within 10 h	[134]
Temperature	Poly(vinyl alcohol)	Glyphosate	The release rate after 13 h is 12%	[128]
Temperature	Phenazine	Clopyralid	At 10, 20, and 30 °C, after 20 h, the release was 49.245%, 53.644%, and 55.797%	[129]
Temperature and pH	Porous carbon nanoparticles	Paraquat	At pH 2.0, the release rate after 24 h is 9.7%, and after 14 days it is 22.8%	[130]
pH	Polyvinyl pyrrolidone	Glyphosate	At pH 6.5, the release amount reaches 74.5% after 24 h	[135]
pH	Tannic acid	Prometryn	At pH 5.5, the release amount over 24 h is 80.5%	[66]
pH	ZIF-67	Pretilachlor and AD-67	The overall release rates of Pretilachlor and AD-67 on the seventh day were 86% and 96%, respectively	[77]
Enzyme	Polyethylenimine	Pendimethalin	The cumulative release rate reached 81.94% in 30 h	[70]

## References

[1] Rojas S., Rodríguez-Diéguez A., Horcajada P. (2022). Metal–organic frameworks in agriculture. ACS Appl. Mater. Interfaces.

[2] Fuglie K. (2021). Climate change upsets agriculture. Nat. Clim. Change.

[3] Kubiak A., Wolna-Maruwka A., Niewiadomska A., Pilarska A.A. (2022). The problem of weed infestation of agricultural plantations vs. the assumptions of the european biodiversity strategy. Agronomy.

[4] Jayasoorya R., Kumar P. (2024). Utilization of biodegradable carrier-based nano herbicide formulations for sustainable weed management in agriculture. Front. Agron..

[5] Goswami L., Kim K.-H., Deep A., Das P., Bhattacharya S.S., Kumar S., Adelodun A.A. (2017). Engineered nano particles: Nature, behavior, and effect on the environment. J. Environ. Manag..

[6] Ofosu R., Agyemang E.D., Márton A., Pásztor G., Taller J., Kazinczi G. (2023). Herbicide resistance: Managing weeds in a changing world. Agronomy.

[7] Pathak V.M., Verma V.K., Rawat B.S., Kaur B., Babu N., Sharma A., Dewali S., Yadav M., Kumari R., Singh S. (2022). Current status of pesticide effects on environment, human health and it’s eco-friendly management as bioremediation: A comprehensive review. Front. Microbiol..

[8] Tyagi P.K., Arya A., Ramniwas S., Tyagi S. (2023). Editorial: Recent trends in nanotechnology in precision and sustainable agriculture. Front. Plant Sci..

[9] Kah M., Beulke S., Tiede K., Hofmann T. (2013). Nanopesticides: State of knowledge, environmental fate, and exposure modeling. Crit. Rev. Environ. Sci. Technol..

[10] Wang D., Saleh N.B., Byro A., Zepp R., Sahle-Demessie E., Luxton T.P., Ho K.T., Burgess R.M., Flury M., White J.C. (2022). Nano-enabled pesticides for sustainable agriculture and global food security. Nat. Nanotechnol..

[11] Jiang X., Yang F., Jia W., Jiang Y., Wu X., Song S., Shen H., Shen J. (2024). Nanomaterials and nanotechnology in agricultural pesticide delivery: A review. Langmuir.

[12] Tao R., You C., Qu Q., Zhang X., Deng Y., Ma W., Huang C. (2023). Recent advances in the design of controlled and sustained-release micro/nanocarriers of pesticide. Environ. Sci. Nano.

[13] Tangamornsuksan W., Lohitnavy O., Sruamsiri R., Chaiyakunapruk N., Scholfield C.N., Reisfeld B., Lohitnavy M. (2019). Paraquat exposure and parkinson’s disease: A systematic review and meta-analysis. Arch. Environ. Occup. Health.

[14] Mohd Ghazi R., Nik Yusoff N.R., Abdul Halim N.S., Wahab I.R.A., Ab Latif N., Hasmoni S.H., Ahmad Zaini M.A., Zakaria Z.A. (2023). Health effects of herbicides and its current removal strategies. Bioengineered.

[15] Dong W., Ren Y., Xue H. (2024). Fabrication and application of carrier-free and carrier-based nanopesticides in pest management. Arch. Insect Biochem. Physiol..

[16] Ghormade V., Deshpande M.V., Paknikar K.M. (2011). Perspectives for nano-biotechnology enabled protection and nutrition of plants. Biotechnol. Adv..

[17] Zhao X., Cui H., Wang Y., Sun C., Cui B., Zeng Z. (2018). Development strategies and prospects of nano-based smart pesticide formulation. J. Agric. Food Chem..

[18] Cheng X., Wang A., Cao L., Cao C., Zhao P., Yu M., Zheng L., Huang Q. (2024). Efficient delivery of the herbicide quinclorac by nanosuspension for enhancing deposition, uptake and herbicidal activity. Pest Manag. Sci..

[19] Kumar A., Kanwar R., Mehta S.K. (2021). Development of phosphatidylcholine/tween 80 based biocompatible clove oil-in-water nanoemulsion as a green nanocarrier for controlled herbicide delivery. Environ. Pollut..

[20] Singh A., Dhiman N., Kar A.K., Singh D., Purohit M.P., Ghosh D., Patnaik S. (2019). Advances in controlled release pesticide formulations: Prospects to safer integrated pest management and sustainable agriculture. J. Hazard. Mater..

[21] Prudnikova S.V., Boyandin A.N., Kalacheva G.S., Sinskey A.J. (2012). Degradable polyhydroxyalkanoates as herbicide carriers. J. Polym. Environ..

[22] Preisler A.C., Pereira A.E., Campos E.V., Dalazen G., Fraceto L.F., Oliveira H.C. (2019). Atrazine nanoencapsulation improves pre-emergence herbicidal activity against bidens pilosa without enhancing long-term residual effect on glycine max. Pest Manag. Sci..

[23] Takeshita V., de Sousa B.T., Preisler A.C., Carvalho L.B., Pereira A.d.E.S., Tornisielo V.L., Dalazen G., Oliveira H.C., Fraceto L.F. (2021). Foliar absorption and field herbicidal studies of atrazine-loaded polymeric nanoparticles. J. Hazard. Mater..

[24] Pereira A.E.S., Grillo R., Mello N.F.S., Rosa A.H., Fraceto L.F. (2014). Application of poly(epsilon-caprolactone) nanoparticles containing atrazine herbicide as an alternative technique to control weeds and reduce damage to the environment. J. Hazard. Mater..

[25] Sousa B., Do Espirito Santo Pereira A., Fraceto L.F., De Oliveira H.C., Dalazen G. (2020). Effectiveness of nanoatrazine in post-emergent control of the tolerant weed *Digitaria insularis*. J. Plant Prot. Res..

[26] Bombo A.B., Pereira A., Lusa M.G., Medeiros O.E., Oliveira J.L., Campos E., Jesus M.B., Oliveira H.C., Fraceto L.F., Mayer J. (2019). A mechanistic view of interactions of a nanoherbicide with target organism. J. Agric. Food Chem..

[27] De Sousa B.T., Do Espirito Santo Pereira A., Fraceto L.F., Oliveira H.C., Dalazen G. (2022). Post-emergence herbicidal activity of nanoatrazine against alternanthera tenella colla plants compared to other weed species. Heliyon.

[28] Diyanat M., Saeidian H. (2019). The metribuzin herbicide in polycaprolactone nanocapsules shows less plant chromosome aberration than non-encapsulated metribuzin. Environ. Chem. Lett..

[29] Takeshita V., Preisler A.C., Munhoz-Garcia G.V., Bragança L., De Pinácio C.W., Oliveira H.C., Tornisielo V.L., Cardoso B.C., Ramalho E.F.B., Pimpinato R.F. (2024). A multi-technique approach for nanoherbicide tracking: Uptake and translocation pathways of metribuzin nanocarrier in weed plants. Environ. Sci. Nano.

[30] Takeshita V., Munhoz-Garcia G.V., Pinácio C.W., Cardoso B.C., Nalin D., Tornisielo V.L., Fraceto L.F. (2022). Availability of metribuzin-loaded polymeric nanoparticles in different soil systems: An important study on the development of safe nanoherbicides. Plants.

[31] Takeshita V., Carvalho L.B., Galhardi J.A., Munhoz-Garcia G.V., Pimpinato R.F., Oliveira H.C., Tornisielo V.L., Fraceto L.F. (2022). Development of a preemergent nanoherbicide: From efficiency evaluation to the assessment of environmental fate and risks to soil microorganisms. ACS Nanosci. Au.

[32] CHEN X., Wang T. (2019). Preparation and characterization of atrazine-loaded biodegradable PLGA nanospheres. J. Integr. Agric..

[33] Schnoor B., Elhendawy A., Joseph S., Putman M., Chacón-Cerdas R., Flores-Mora D., Bravo-Moraga F., Gonzalez-Nilo F., Salvador-Morales C. (2018). Engineering atrazine loaded poly (lactic-co-glycolic acid) nanoparticles to ameliorate environmental challenges. J. Agric. Food Chem..

[34] Kannamreddy V., Chinnamuthu C.R., Marimuthu S., Bharathi C. (2021). Synthesizing nanoencapsulated sulfentrazone herbicide and optimizing time and dose for season long weed management in irrigated blackgram (*Vigna mungo* L.). Legume Res..

[35] Mahmoudian M., Torbati S., AliMirzayi N., Nozad E., Kochameshki M.G., Shokri A. (2020). Preparation and investigation of poly(methylmethacrylate) nano-capsules containing haloxyfop-r-methyl and their release behavior. J. Environ. Sci. Health B.

[36] Takeshita V., Oliveira F.F., Garcia A., Zuverza-Mena N., Tamez C., Cardoso B.C., De Pinácio C.W., Steven B., LaReau J.C., Astete C.E. (2024). Delivering metribuzin from biodegradable nanocarriers: Assessing herbicidal effects for soybean plant protection and weed control. Environ. Sci. Nano.

[37] Diyanat M., Saeidian H., Baziar S., Mirjafary Z. (2019). Preparation and characterization of polycaprolactone nanocapsules containing pretilachlor as a herbicide nanocarrier. Environ. Sci. Pollut. Res. Int..

[38] Grillo R., Rosa A.H., Fraceto L.F. (2014). Poly(ε-caprolactone) nanocapsules carrying the herbicide atrazine: Effect of chitosan-coating agent on physico-chemical stability and herbicide release profile. Int. J. Environ. Sci. Technol..

[39] Sousa B.T., Carvalho L.B., Preisler A.C., Saraiva-Santos T., Oliveira J.L., Verri W.A., Dalazen G., Fraceto L.F., Oliveira H. (2024). Chitosan coating as a strategy to increase postemergent herbicidal efficiency and alter the interaction of nanoatrazine with bidens pilosa plants. ACS Appl. Mater. Interfaces.

[40] Khan B.A., Nadeem M.A., Iqbal M., Yaqoob N., Javaid M.M., Maqbool R., Elnaggar N., Oraby H. (2023). Chitosan nanoparticles loaded with mesosulfuron methyl and mesosulfuron methyl + florasulam + MCPA isooctyl to manage weeds of wheat (*Triticum aestivum* L.). Green Process. Synth..

[41] Ghaderpoori M., Jafari A., Nazari E., Rashidipour M., Nazari A., Chehelcheraghi F., Kamarehie B., Rezaee R. (2020). Preparation and characterization of loaded paraquat- polymeric chitosan/xantan/tripolyphosphate nanocapsules and evaluation for controlled release. J. Environ. Health Sci. Eng..

[42] Babaei S., Kahrizi D., Nosratti I., Karimi N., Arkan E., Tahir M.B. (2022). Preparation and characterization of chloridazon-loaded alginate/chitosan nanocapsules. Cell Mol. Biol..

[43] Dos Santos Silva M., Cocenza D.S., Grillo R., De Melo N.F.S., Tonello P.S., De Oliveira L.C., Cassimiro D.L., Rosa A.H., Fraceto L.F. (2011). Paraquat-loaded alginate/chitosan nanoparticles: Preparation, characterization and soil sorption studies. J. Hazard. Mater..

[44] Maruyama C.R., Guilger M., Pascoli M., Bileshy-José N., Abhilash P.C., Fraceto L.F., de Lima R. (2016). Nanoparticles based on chitosan as carriers for the combined herbicides imazapic and imazapyr. Sci. Rep..

[45] Rashidipour M., Maleki A., Kordi S., Birjandi M., Pajouhi N., Mohammadi E., Heydari R., Rezaee R., Rasoulian B., Davari B. (2019). Pectin/chitosan/tripolyphosphate nanoparticles: Efficient carriers for reducing soil sorption, cytotoxicity, and mutagenicity of paraquat and enhancing its herbicide activity. J. Agric. Food Chem..

[46] Munhoz-Garcia G.V., Takeshita V., Oliveira J.L., Vecchia B.D., Nalin D., De Werk Pinácio C., De Oliveira A.L.C., Cardoso B.C., Tornisielo V.L., Fraceto L.F. (2025). Nanobased natural polymers as a carrier system for glyphosate: An interesting approach aimed at sustainable agriculture. J. Agric. Food Chem..

[47] Artusio F., Casà D., Granetto M., Tosco T., Pisano R. (2021). Alginate nanohydrogels as a biocompatible platform for the controlled release of a hydrophilic herbicide. Processes.

[48] Flores-Céspedes F., Daza-Fernández I., Villafranca-Sánchez M., Fernández-Pérez M., Morillo E., Undabeytia T. (2017). Lignin and ethylcellulose in controlled release formulations to reduce leaching of chloridazon and metribuzin in light-textured soils. J. Hazard. Mater..

[49] Carvalho L.B., Godoy I.S., Preisler A.C., de Freitas Proença P.L., Saraiva-Santos T., Verri W.A., Oliveira H.C., Dalazen G., Fraceto L.F. (2023). Pre-emergence herbicidal efficiency and uptake of atrazine-loaded zein nanoparticles: A sustainable alternative to weed control. Environ. Sci. Nano.

[50] Heydari M., Yousefi A.R., Nikfarjam N., Rahdar A., Kyzas G.Z., Bilal M. (2021). Plant-based nanoparticles prepared from protein containing tribenuron-methyl: Fabrication, characterization, and application. Chem. Biol. Technol. Agric..

[51] Tang G., Tian Y., Gao Y., Zhou Z., Chen X., Li Y., Yu X., Wang H., Li X., Cao Y. (2022). Supramolecular self-assembly of herbicides with reduced risks to the environment. ACS Nano.

[52] Nadiminti P.P., Sharma H., Kada S.R., Pfeffer F.M., Dell L.A.O., Cahill D.M. (2019). Use of Mg–Al nanoclay as an efficient vehicle for the delivery of the herbicide 2,4-dichlorophenoxyacetic acid. ACS Sustain. Chem. Eng..

[53] Ghazali S.A.I.S.M., Sarijo S.H., Hussein M.Z. (2021). New synthesis of binate herbicide-interleaved anionic clay material: Synthesis, characterization and simultaneous controlled-release properties. J. Porous Mater..

[54] Khatem R., Celis R., Hermosín M.C. (2019). Cationic and anionic clay nanoformulations of imazamox for minimizing environmental Risk. Appl. Clay Sci..

[55] Granetto M., Serpella L., Fogliatto S., Re L., Bianco C., Vidotto F., Tosco T. (2022). Natural clay and biopolymer-based nanopesticides to control the environmental spread of a soluble herbicide. Sci. Total Environ..

[56] Zhong B., Wang S., Dong H., Luo Y., Jia Z., Zhou X., Chen M., Xie D., Jia D. (2017). Halloysite tubes as nanocontainers for herbicide and its controlled release in biodegradable poly(vinyl alcohol)/starch film. J. Agric. Food Chem..

[57] Zhang L., Chen C., Zhang G., Liu B., Wu Z., Cai D. (2020). Electrical-driven release and migration of herbicide using a gel-based nanocomposite. J. Agric. Food Chem..

[58] Zha X., Hou X., Li Q., Nan H., Ge F., Liu Y., Li F., Zhang D., Tian J. (2022). Loading glyphosate in attapulgite and sodium alginate hydrogels to construct pH-responsive controlled release microsphere for enhanced soil sustained release. ACS Agric. Sci. Technol..

[59] Jain S.K., Dutta A., Kumar J., Shakil N.A. (2020). Preparation and characterization of dicarboxylic acid modified starch-clay composites as carriers for pesticide delivery. Arab. J. Chem..

[60] Wang X., Hou X., Zou P., Huang A., Zhang M., Ma L. (2022). Cationic starch modified bentonite-alginate nanocomposites for highly controlled diffusion release of pesticides. Int. J. Biol. Macromol..

[61] Giroto A., Campos A., Pereira E., Cruz C., Marconcini J., Ribeiro C. (2014). Study of a nanocomposite starch-clay for slow-release of herbicides: Evidence of synergistic effects between the biodegradable matrix and exfoliated clay on herbicide release control. J. Appl. Polym. Sci..

[62] Kong F., Zhang Q., Xie Y., Ding J., Zhao H., Zhang Z., Ma Z., Cong H., Meng Z. (2023). Controlled release of herbicides through glyphosate intercalated layered double hydroxides and enhancement of anti-scouring ability via poly-l-aspartic acid and chitosan modification. Int. J. Biol. Macromol..

[63] Wilpiszewska K., Spychaj T., Paździoch W. (2016). Carboxymethyl starch/montmorillonite composite microparticles: Properties and controlled release of isoproturon. Carbohydr. Polym..

[64] Del Carmen Galán-Jiménez M., Morillo E., Bonnemoy F., Mallet C., Undabeytia T. (2020). A Sepiolite-based formulation for slow release of the herbicide mesotrione. Appl. Clay Sci..

[65] Tan D., Yuan P., Annabi-Bergaya F., Liu D., He H. (2015). Methoxy-modified kaolinite as a novel carrier for high-capacity loading and controlled-release of the herbicide amitrole. Sci. Rep..

[66] Teng G., Chen C., Ma X., Mao H., Yuan X., Xu H., Wu Z., Zhang J. (2024). Spherical assembly of halloysite clay nanotubes as a general reservoir of hydrophobic pesticides for pH-responsive management of pests and weeds. Small.

[67] Cao L., Zhou Z., Niu S., Cao C., Li X., Shan Y., Huang Q. (2017). Positive-charge functionalized mesoporous silica nanoparticles as nanocarriers for controlled 2,4-dichlorophenoxy acetic acid sodium salt release. J. Agric. Food Chem..

[68] Shan Y., Cao L., Xu C., Zhao P., Cao C., Li F., Xu B., Huang Q. (2019). Sulfonate-functionalized mesoporous silica nanoparticles as carriers for controlled herbicide diquat dibromide release through electrostatic interaction. Int. J. Mol. Sci..

[69] Deng X., Li J.-Q., Yi J.-M., Lian R.-J., Zhang Z.-Y., Li J.-H., He S., Bai L.-Y. (2023). A pH-responsive MOF-functionalized hollow mesoporous silica controlled herbicide delivery system exhibits enhanced activity against ACCase-herbicide-resistant weeds. Pest Manag. Sci..

[70] Liang Y., Guo M., Fan C., Dong H., Ding G., Zhang W., Tang G., Yang J., Kong D., Cao Y. (2017). Development of novel urease-responsive pendimethalin microcapsules using silica-IPTS-PEI as controlled release carrier materials. ACS Sustain. Chem. Eng..

[71] Wang M., Lou J., Chen Y., Yang L., Wang H. (2023). Preparation and properties of photoresponsive pendimethalin@silica-cinnamamide/γ-CD microspheres for pesticide controlled release. J. Agric. Food Chem..

[72] Mejías F.J.R., Trasobares S., Varela R.M., Molinillo J.M.G., Calvino J.J., Macías F.A. (2021). One-step encapsulation of ortho-disulfides in functionalized zinc MOF enabling metal-organic frameworks in agriculture. ACS Appl. Mater. Interfaces.

[73] Ji Y., Ma S., Lv S., Wang Y., Lü S., Liu M. (2021). Nanomaterials for targeted delivery of agrochemicals by an all-in-one combination strategy and deep learning. ACS Appl. Mater. Interfaces.

[74] Ren L., Li W., Li Q., Zhang D., Fang W., Yan D., Li Y., Wang Q., Jin X., Cao A. (2022). metolachlor metal–organic framework nanoparticles for reducing leaching, ecotoxicity and improving bioactivity. Pest Manag. Sci..

[75] Sierra-Serrano B., García-García A., Hidalgo T., Ruiz-Camino D., Rodríguez-Diéguez A., Amariei G., Rosal R., Horcajada P., Rojas S. (2022). Copper glufosinate-based metal–organic framework as a novel multifunctional agrochemical. ACS Appl. Mater. Interfaces.

[76] Lee S., Wang G., Ji N., Zhang M., Wang D., Sun L., Meng W., Zheng Y., Li Y., Wu Y. (2021). Synthesis, characterizations and kinetics of MOF-5 as herbicide vehicle and its controlled release in PVA/ST biodegradable composite membranes. Z. Für Anorg. Und Allg. Chem..

[77] Guo K., Deng X., Peng Y., Yang N., Qian K., Bai L. (2023). A MOF based pH-responsive dual controlled release system for herbicide pretilachlor and safener AD-67 delivery enhances the herbicidal efficacy and reduces the side effects. Environ. Sci. Nano.

[78] Iyarin T.M.E., Aravind Kumar B.N., Babu R., Nirmalnath P.J., Hebsur N.S., Halli H.M., Govindasamy P., Senthamil E., Sannagoudar M.S., Palsaniya D.R. (2024). Nanocomposite based slow release atrazine effectively controlled striga asiatica incidence, and enhanced sugarcane yield. Sci. Rep..

[79] Yang J., Zang W., Zhang Z., Wang P., Yang Q. (2019). The enhanced and tunable sustained release of pesticides using activated carbon as a carrier. Materials.

[80] Tang G., Wang J., Xiao J., Liu Y., Huang Y., Zhou Z., Zhang X., Hu G., Yan W., Cao Y. (2024). Amphiphilic cationic carbon dots for efficient delivery of light-dependent herbicide. Adv. Sci..

[81] Deng X., Zhao P., Xie Y., Bai L. (2023). Self-assembled sphere covalent organic framework with enhanced herbicidal activity by loading cyhalofop-butyl. J. Agric. Food Chem..

[82] Sinha V.R., Bansal K., Kaushik R., Kumria R., Trehan A. (2004). Poly-ϵ-caprolactone microspheres and nanospheres: An overview. Int. J. Appl. Pharm..

[83] Grillo R., dos Santos N.Z.P., Maruyama C.R., Rosa A.H., de Lima R., Fraceto L.F. (2012). Poly(ε-Caprolactone) nanocapsules as carrier systems for herbicides: Physico-chemical characterization and genotoxicity evaluation. J. Hazard. Mater..

[84] Byun Y., Hwang J.B., Bang S.H., Darby D., Cooksey K., Dawson P.L., Park H.J., Whiteside S. (2011). Formulation and characterization of α-tocopherol loaded poly ɛ-caprolactone (PCL) nanoparticles. Lebensm.-Wiss. Technol..

[85] Oliveira H.C., Stolf-Moreira R., Martinez C.B.R., Sousa G.F.M., Grillo R., Jesus M.B., Fraceto L.F. (2015). Evaluation of the side effects of poly(epsilon-caprolactone) nanocapsules containing atrazine toward maize plants. Front. Chem..

[86] Azwa Z.N., Yousif B.F., Manalo A.C., Karunasena W. (2013). A review on the degradability of polymeric composites based on natural fibres. Mater. Des..

[87] Volova T., Shumilova A., Zhila N., Sukovatyi A., Shishatskaya E., Thomas S. (2020). Efficacy of slow-release formulations of metribuzin and tribenuron methyl herbicides for controlling weeds of various species in wheat and barley stands. ACS Omega.

[88] Wang X., Tarahomi M., Sheibani R., Xia C., Wang W. (2023). Progresses in lignin, cellulose, starch, chitosan, chitin, alginate, and gum/carbon nanotube (nano)composites for environmental applications: A review. Int. J. Biol. Macromol..

[89] Smola-Dmochowska A., Lewicka K., Macyk A., Rychter P., Pamuła E., Dobrzyński P. (2023). Biodegradable polymers and polymer composites with antibacterial properties. Int. J. Mol. Sci..

[90] Egorov A.R., Kirichuk A.A., Rubanik V.V., Rubanik V.V., Tskhovrebov A.G., Kritchenkov A.S. (2023). Chitosan and its derivatives: Preparation and antibacterial properties. Materials.

[91] Pontes M., Antunes D., De Oliveira I.P., Forini M., Santos J., Arruda G., Caires A., Santiago E.F., Grillo R. (2021). Chitosan/tripolyphosphate nanoformulation carrying paraquat: Insights of its enhanced herbicidal activity. Environ. Sci. Nano.

[92] Grillo R., Pereira A.E.S., Nishisaka C.S., Lima R., Oehlke K., Greiner R., Fraceto L.F. (2014). Chitosan/tripolyphosphate nanoparticles loaded with paraquat herbicide: An environmentally safer alternative for weed control. J. Hazard. Mater..

[93] Grillo R., Clemente Z., De Oliveira J.L., Campos E.V.R., Chalupe V.C., Jonsson C.M., De Lima R., Sanches G., Nishisaka C.S., Rosa A.H. (2015). Chitosan nanoparticles loaded the herbicide paraquat: The influence of the aquatic humic substances on the colloidal stability and toxicity. J. Hazard. Mater..

[94] Satchanska G., Davidova S., Petrov P.D. (2024). Natural and synthetic polymers for biomedical and environmental applications. Polymers.

[95] Zhang Z., Yang N., Yu J., Jin S., Shen G., Chen H., Yuzhen N., Xiang D., Qian K. (2023). Research Progress of a Pesticide Polymer-Controlled Release System Based on Polysaccharides. Polymers.

[96] Pal A., Kaur P., Dwivedi N., Rookes J., Bohidar H.B., Yang W., Cahill D.M., Manna P.K. (2022). Clay-nanocomposite based smart delivery systems: A promising tool for sustainable farming. ACS Agric. Sci. Technol..

[97] Choy J.-H., Choi S.-J., Oh J.-M., Park T. (2007). Clay minerals and layered double hydroxides for novel biological applications. Appl. Clay Sci..

[98] Dähn R., Scheidegger A.M., Manceau A., Schlegel M.L., Baeyens B., Bradbury M.H., Chateigner D. (2003). Structural evidence for the sorption of Ni(II) atoms on the edges of montmorillonite clay minerals. A polarized x-ray absorption fine structure study. Geochim. Cosmochim. Acta.

[99] Natarelli C.V.L., Claro P.I.C., Miranda K.W.E., Ferreira G.M.D., Oliveira J.E., Marconcini J.M. (2019). 2,4-Dichlorophenoxyacetic acid adsorption on montmorillonite organoclay for controlled release applications. SN Appl. Sci..

[100] Jia X., Yan Y., Zhang K., Wang C., You X., Yang S., Wang J., Zhang B., Wang Y., Xie J. (2023). Glufosinate ammonium-loaded halloysite nanotubes for slow-release weeding polymer mulch films. ACS Appl. Nano Mater..

[101] Gupta P., Sharma V., Nagpal G. (2025). A comprehensive review of nano clay: From development and applications to research opportunities. Environ. Prog. Sustain. Energy.

[102] Zhang T., Xu Z., Xu Z., Ma Y., Niu Z., Chen J., Zhang M., Shi F. (2025). Progress on layered double hydroxides as green materials in sustainable agricultural production. Adv. Environ. Res..

[103] Yan G., Huang Q., Zhao S., Xu Y., He Y., Nikolic M., Nikolic N., Liang Y., Zhu Z. (2024). Silicon nanoparticles in sustainable agriculture: Synthesis, absorption, and plant stress alleviation. Front. Plant Sci..

[104] Xu C., Lei C., Yu C. (2019). Mesoporous silica nanoparticles for protein protection and delivery. Front. Chem..

[105] Yang Y., Zhang M., Song H., Yu C. (2020). Silica-based nanoparticles for biomedical applications: From nanocarriers to biomodulators. Acc. Chem. Res..

[106] Zhang J., Karmakar S., Yu M., Mitter N., Zou J., Yu C. (2014). Synthesis of silica vesicles with controlled entrance size for high loading, sustained release, and cellular delivery of therapeutical proteins. Small.

[107] Farha O.K., Eryazici I., Jeong N.C., Hauser B.G., Wilmer C.E., Sarjeant A.A., Snurr R.Q., Nguyen S.T., Yazaydın A.Ö., Hupp J.T. (2012). Metal–organic framework materials with ultrahigh surface areas: Is the sky the limit?. J. Am. Chem. Soc..

[108] Chen Z., Jiang H., Li M., Keeffe M.O., Eddaoudi M. (2020). Reticular chemistry 3.2: Typical minimal edge-transitive derived and related nets for the design and synthesis of metal–organic frameworks. Chem. Rev..

[109] Jeong C., Ansari M.Z., Anwer A.H., Kim S.-H., Nasar A., Shoeb M., Mashkoor F. (2023). Areview on metal-organic frameworks for the removal of hazardous environmental contaminants. Sep. Purif. Technol..

[110] Mondol M.M.H., Jhung S.H. (2021). Adsorptive removal of pesticides from water with metal-organic framework-based materials. Chem. Eng. J..

[111] Liu Y.-Q., Song C., Ding G., Yang J., Wu J., Wu G., Zhang M.Z., Song C., Guo L.-P., Qin J. (2022). High-performance functional Fe-MOF for removing aflatoxin B1 and other organic pollutants. Adv. Mater. Interfaces.

[112] Zhao Y., Zuo X., Lu X., Li Z., Gao F. (2022). Hierarchical porous hollow N-doped Cu-based MOF derivatives as highly sensitive electrochemical sensing platform for pesticides detection. Sens. Actuators B.

[113] Sun D.W., Huang L., Pu H., Ma J. (2021). Introducing reticular chemistry into agrochemistry. Chem. Soc. Rev..

[114] Bruneau M., Bennici S., Brendle J., Dutournie P., Limousy L., Pluchon S. (2019). Systems for stimuli-controlled release: Materials and applications. J. Control. Release.

[115] Sohrabi H., Salahshour Sani P., Orooji Y., Majidi M.R., Yoon Y., Khataee A. (2022). MOF-based sensor platforms for rapid detection of pesticides to maintain food quality and safety. Food Chem. Toxicol..

[116] Tao Y., Fang F., Lv Q., Qin W., He X., Zhang Y., Zhou Y., Li X., Li J. (2022). Highly efficient removal of glyphosate from water by hierarchical-pore UiO-66: Selectivity and effects of natural water particles. J. Environ. Manage..

[117] Zhang Y., Lei Y., Yan T., Liao Y., Han G. (2024). Efficient adsorption removal of 2,4-dichlorophenoxyacetic acid using amine-functionalized metal–organic frameworks (MOFs): Performance and mechanisms. Sep. Purif. Technol..

[118] Vikrant K., Kumar V., Kim K.H., Kukkar D. (2017). Metal–organic frameworks (MOFs): Potential and challenges for capture and abatement of ammonia. J. Mater. Chem..

[119] Fang Q., Wang J., Gu S., Kaspar R.B., Zhuang Z., Zheng J., Guo H., Qiu S., Yan Y. (2015). 3D porous crystalline polyimide covalent organic frameworks for drug delivery. J. Am. Chem. Soc..

[120] Diercks C.S., Yaghi O.M. (2017). The atom, the molecule, and the covalent organic framework. Science.

[121] Nguyen M.N. (2021). Potential use of silica-rich biochar for the formulation of adaptively controlled release fertilizers: A mini review. J. Clean. Prod..

[122] Dai Y., Zhang N., Xing C., Cui Q., Sun Q. (2019). The adsorption, regeneration and engineering applications of biochar for removal organic pollutants: A review. Chemosphere.

[123] Gougherty A.V., Davies T.J. (2021). Towards a phylogenetic ecology of plant pests and pathogens. Philos. Trans. R. Soc..

[124] Wang Y., Peng Z., Yang Y., Li Z., Wen Y., Liu M., Li S., Su L., Zhou Z., Zhu Y. (2022). Auricularia auricula biochar supported γ-FeOOH nanoarrays for electrostatic self-assembly and pH-responsive controlled release of herbicide and fertilizer. Chem. Eng. J..

[125] Shen M., Liu S., Jiang C., Zhang T., Chen W. (2023). Recent advances in stimuli-response mechanisms of nano-enabled controlled-release fertilizers and pesticides. Eco-Environ. Health.

[126] Chen C., Zhang G., Dai Z., Xiang Y., Liu B., Bian P., Zheng K., Wu Z., Cai D. (2018). Fabrication of light-responsively controlled-release herbicide using a nanocomposite. Chem. Eng. J..

[127] Shan P., Lu Y., Lu W., Yin X., Liu H., Li D., Lian X., Wang W., Li Z., Li Z. (2022). Biodegradable and light-responsive polymeric nanoparticles for environmentally safe herbicide delivery. ACS Appl. Mater. Interface.

[128] Chi Y., Zhang G., Xiang Y., Cai D., Wu Z. (2017). Fabrication of a temperature-controlled-release herbicide using a nanocomposite. ACS Sustain. Chem. Eng..

[129] Xiao Y., Wu C., Liu Y., Zhou L., Wu S., Yin Q. (2024). Biocompatible nano-cocrystal engineering for targeted herbicide delivery: Enhancing efficacy through stimuli-responsive release and reduced environmental losses. ACS Appl. Mater. Interfaces.

[130] Dong J., Liu X., Chen Y., Yang W., Du X. (2021). User-safe and efficient chitosan-gated porous carbon nanopesticides and nanoherbicides. J. Colloid. Interface Sci..

[131] Atta S., Bera M., Paul A., Singh P. (2015). Nano-pesticide formulation based on fluorescent organic photoresponsive nanoparticles: For controlled release of 2,4-D and real time monitoring of morphological changes induced by 2,4-D in plant systems. RSC Adv..

[132] Shan P., Li D., Lu W., Lu W., Yin X., Lian X., Lu Y., Qi Y., Zhang M., Du K. (2023). Photo-degradable functional polyesters from an o-nitrobenzyl dithiol: Synthesis and applications in herbicide delivery. ACS Appl. Polym. Mater..

[133] Feng S., Wang J., Zhang L., Chen Q., Yue W., Ke N., Xie H. (2020). Coumarin-containing light-responsive carboxymethyl chitosan micelles as nanocarriers for controlled release of pesticide. Polymers.

[134] Gao C., Huang Q., Lan Q., Feng Y., Tang F., Hoi M., Zhang J., Lee S., Wang R. (2018). A User-friendly herbicide derived from photo-responsive supramolecular vesicles. Nat. Commun..

[135] Zeng Y., Li X., Chen F., Ye H., Rong K., Ran Z., Liu B., Pan Z., Xie X., Tang J. (2024). Biomineralization-inspired porous calcium carbonate microspheres as a controlled release system of herbicides/pesticides. J. Environ. Chem. Eng..

[136] Younis S.A., Kim K.-H., Shaheen S.M., Antoniadis V., Tsang Y.F., Rinklebe J., Deep A., Brown R.J. (2021). Advancements of nanotechnologies in crop promotion and soil fertility: Benefits, life cycle assessment, and legislation policies. Renew. Sustain. Energy Rev..

[137] Rajput V., Minkina T., Mazarji M., Shende S., Sushkova S., Mandzhieva S., Burachevskaya M., Chaplygin V., Singh A., Jatav H. (2020). Accumulation of nanoparticles in the soil-plant systems and their effects on human health. Ann. Agric. Sci..

[138] Thuesombat P., Hannongbua S., Akasit S., Chadchawan S. (2014). Effect of silver nanoparticles on rice (*Oryza sativa* L. *cv. KDML* 105) seed germination and seedling growth. Ecotoxicol. Environ. Saf..

[139] Juárez-Maldonado A., Tortella G., Rubilar O., Fincheira P., Benavides-Mendoza A. (2021). Biostimulation and toxicity: The magnitude of the impact of nanomaterials in microorganisms and plants. J. Adv. Res..

[140] Yang X., He Q., Guo F., Sun X., Zhang J., Chen Y. (2021). Impacts of carbon-based nanomaterials on nutrient removal in constructed wetlands: Microbial community structure, enzyme activities, and metabolism process. J. Hazard. Mater..

[141] Da Costa M.V.J., Sharma P.K. (2016). Effect of copper oxide nanoparticles on growth, morphology, photosynthesis, and antioxidant response in Oryza sativa. Photosynthetica..

[142] Rajput V.D., Minkina T., Sushkova S., Tsitsuashvili V., Mandzhieva S., Gorovtsov A., Nevidomskyaya D., Gromakova N. (2018). Effect of nanoparticles on crops and soil microbial communities. J. Soils Sediments.

[143] Huang Y., Zhao L., Keller A.A. (2017). Interactions, Transformations, and Bioavailability of Nano-Copper Exposed to Root Exudates. Environ. Sci. Technol..

[144] Zhou J., Liu G., Guo Z., Wang M., Qi C., Chen G., Huang X., Yan S., Xu D. (2023). Stimuli-responsive pesticide carriers based on porous nanomaterials: A review. Chem. Eng. J..

[145] Gladkova M.M., Terekhova V.A. (2013). Engineered nanomaterials in soil: Sources of entry and migration pathways. Mosc. Univ. Soil Sci. Bull..

[146] Zhang P., Guo Z., Ullah S., Melagraki G., Afantitis A., Lynch I. (2021). Nanotechnology and artificial intelligence to enable sustainable and precision agriculture. Nat. Plants.

[147] Shang Y., Hasan M.K., Ahammed G.J., Li M., Yin H., Zhou J. (2019). Applications of Nanotechnology in Plant Growth and Crop Protection: A Review. Molecules.

[148] Chaud M., Souto E.B., Zielinska A., Severino P., Batain F., Oliveira-Junior J., Alves T. (2021). Nanopesticides in Agriculture: Benefits and Challenge in Agricultural Productivity, Toxicological Risks to Human Health and Environment. Toxics.

[149] Schwab F. (2022). Opportunities and Limitations of Nanoagrochemicals. Helv. Chim. Acta.

[150] Santos P.A., Biraku X., Nielsen E., Ozketen A.C., Ozketen A.A., Hakki E.E. (2025). Agricultural nanotechnology for a safe and sustainable future: Current status, challenges, and beyond. J. Sci. Food Agric..

